# Arginase 1 deletion in myeloid cells affects the inflammatory response in allergic asthma, but not lung mechanics, in female mice

**DOI:** 10.1186/s12890-017-0490-7

**Published:** 2017-11-28

**Authors:** Roy H. E. Cloots, Selvakumari Sankaranarayanan, Matthew E. Poynter, Els Terwindt, Paul van Dijk, Wouter H. Lamers, S. Eleonore Köhler

**Affiliations:** 1 Department of Anatomy & Embryology and NUTRIM School of Nutrition and Translational Research in Metabolism, Maastricht University, P.O. Box 616, 6200 MD Maastricht, The Netherlands; 2 Tytgat Institute for Liver and Intestinal Research, Academic Medical Center, Amsterdam, The Netherlands; 3 Division of Pulmonary Disease and Critical Care, Department of Medicine, College of Medicine, University of Vermont, Burlington, VT USA

**Keywords:** Arginase1, Airway hyperresponsiveness, Inflammation

## Abstract

**Background:**

(Over-)expression of arginase may limit local availability of arginine for nitric oxide synthesis. We investigated the significance of arginase1 (ARG1) for the development of airway hyperresponsiveness (AHR) and lung inflammation in female mice with ovalbumin (OVA)-induced allergic asthma.

**Methods:**

*Arg1* was ablated in the lung by crossing *Arg1* ^*fl/fl*^ and *Tie2Cre* ^*tg/−*^ mice. OVA sensitization and challenge were conducted, and AHR to methacholine was determined using the Flexivent system. Changes in gene expression, chemokine and cytokine secretion, plasma IgE, and lung histology were quantified using RT-qPCR, ELISA, and immunohistochemistry, respectively.

**Results:**

*Arg1* ablation had no influence on the development of OVA-induced AHR, but attenuated OVA-induced increases in expression of *Arg2* and *Nos2, Slc7a1, Slc7a2,* and *Slc7a7* (arginine transporters)*, Il4, Il5* and *Il13* (T_H_2-type cytokines)*, Ccl2* and *Ccl11* (chemokines)*, Ifng* (T_H_1-type cytokine), *Clca3* and *Muc5ac* (goblet cell markers), and OVA-specific IgE. Pulmonary IL-10 protein content increased, but IL-4, IL-5, IL-13, TNFα and IFNγ content*,* and lung histopathology, were not affected. *Arg1* elimination also decreased number and tightness of correlations between adaptive changes in lung function and inflammatory parameters in OVA/OVA-treated female mice. OVA/OVA-treated female mice mounted a higher OVA-IgE response than males, but the correlation between lung function and inflammation was lower. *Arg1*-deficient OVA/OVA-treated females differed from males in a more pronounced decline of arginine-metabolizing and -transporting genes, higher plasma arginine levels, a smaller OVA-specific IgE response, and no improvement of peripheral lung function.

**Conclusion:**

Complete ablation of *Arg1* in the lung affects mRNA abundance of arginine-transporting and -metabolizing genes, and pro-inflammatory genes, but not methacholine responsiveness or accumulation of inflammatory cells.

**Electronic supplementary material:**

The online version of this article (10.1186/s12890-017-0490-7) contains supplementary material, which is available to authorized users.

## Background

Allergic asthma is a prevalent disease characterized by airway inflammation, hyperresponsiveness, and eventually remodeling. Development of allergic asthma starts upon binding of inhaled allergens to their complementary IgEs, which activates IgE receptor-bearing mast cells and basophils. These cells, in turn, activate an inflammatory cascade that causes lung infiltration with eosinophils, neutrophils, alternatively activated (M2) macrophages and (T_H_2) lymphocytes. The inflammatory response is typically accompanied by mucus production, mucosal edema, and smooth-muscle hyperresponsiveness, which all contribute to the development of airway hyperresponsiveness (AHR) upon exposure to e.g. histamine and methacholine [1, 2]. Repeated episodes of allergic inflammation eventually lead to fibrosis and wall thickening in large and small airways (“airway remodeling”).

Adult women suffer more often and more severely from asthma than men do (for a recent review, see: [3]). Female (BALB/c) mice develop a more severe airway inflammation with more myeloid dendritic cells, effector T cells, alternatively activated macrophages, eosinophils, and higher concentrations of IgE and cytokines than male mice in ovalbumin (OVA)-induced allergic asthma models [4–6]. Male (C57BL/6) [7, 8] and progesterone-treated female mice [9], on the other hand, develop stronger AHR due to the relaxing effect of estrogens on airway smooth muscle [8, 10, 11]. Taken together, these data reveal a sex difference in mice with respect to the respiratory and immune responses to allergens (for a recent review, see [12]).

Depending on the pro-inflammatory agent, alveolar macrophages can develop into classically activated (M1) macrophages, alternatively activated M2) macrophages, and intermediate forms [13]. Alternatively activated lung macrophages appear to be major players in the development of airway inflammation of OVA-sensitized and -challenged (OVA/OVA) mice, and are more prevalent in female than in male mice [14]. The *arginase1* (*Arg1*) gene is highly expressed in the cytoplasm of M2 macrophages [13, 15]. Accordingly, OVA-induced *Arg1* expression is present in cells with the morphological characteristics of macrophages [16, 17]. The prominence of ARG1-containing macrophages underlies the hypothesis that arginine availability plays a key role in the development and presentation of asthma [18–20].

Arginase and nitric oxide synthase (NOS) share, and compete for the substrate L-arginine. Since NO relaxes the smooth-muscle cells of the bronchi and blood vessels, insufficient NO production may underlie AHR [4]. A high concentration of NO, however, promotes inflammation, mucosal swelling, and mucus secretion in the lung [21, 22]. Although the NOS enzymes have a ~500-fold higher affinity for arginine than arginase, the V_max_ of arginase is ~1000-fold higher than that of NOS, which makes regional substrate depletion of arginine possible [21, 23]. Inhibition of arginine uptake into macrophages by ablation of the arginine transporter *Cat2* [24] and excess cationic proteins, such as eosinophil-derived major basic protein (MBP) [25], also inhibits NO production, whereas pharmacological inhibition of arginase activity attenuates AHR, *Arg1* expression, cell number in bronchoalveolar lavage fluid, and expression of the inflammatory markers IL-4, IL-5, IL-13, and NOS2 [26–29], and induces NO-mediated smooth-muscle relaxation [30]. Furthermore, supplementation of arginine mitigates the inflammatory airway response, increases arginase expression and activity, and elevates NOx levels [31, 32]. These findings suggest that arginase-mediated differences in substrate availability for NO synthesis may determine the clinical presentation of allergic asthma.

In an earlier study, we reported that *Arg1* ablation in macrophages improved peripheral lung function and decreased mRNA expression of arginine-metabolizing and -transporting genes in OVA/OVA-treated male C57Bl/6 mice, but had no effect on airway inflammation [33]. In a similar study with *Arg1* ^*fl/fl*^ */Tie2Cre* ^*tg/−*^ mice, in which *Arg1* exons 7 and 8 instead of exon 4 were flanked by *loxP* sites, Barron et al. also reported the lack of an effect of *Arg1* elimination in myeloid cells on allergic lung inflammation, but found no effect on peripheral lung mechanics [34]. However, these authors did not differentiate between male and female mice. In the present study, we examined the role of ARG1 on lung function and inflammation in OVA/OVA-treated female *Arg1* ^*fl/fl*^ */Tie2Cre* ^*tg/−*^ mice. We report that *Arg1* ablation in the lung of female C57Bl/6 mice did not protect peripheral lung mechanics, as in male mice, but did cause a smaller increase in the expression of L-arginine-metabolizing enzymes and transporters, and of inflammatory cytokines and chemokines.

## Methods

### Generation of transgenic mice and husbandry

All animal experiments were approved by the Committee for Animal Care and Use of Maastricht University (DEC2005-146). The generation of the transgenic mice was described before [33]. In brief: exon 4 of the mouse *Arg1* gene was flanked with *loxP* sites [33] and offspring were genotyped with primers *Arg1-F1* and *Arg1-R1* (Additional file 1: Table S1). To ablate the floxed *Arg1* allele (*Arg1* ^*fl*^) specifically in macrophages, *Arg1* ^*fl/fl*^ mice were crossed with either LysM-Cre [35] or Tie2-Cre mice [36]. The resulting *Arg* ^*fl/fl*^ */LysM-Cre* ^*tg/−*^ (Arg1-KO^LysM^) mice and their *Arg* ^*fl/fl*^ */LysM-Cre* ^*−/−*^ littermates (Arg1-Con) or *Arg* ^*fl/fl*^ */Tie2-Cre* ^*tg/−*^ (Arg1- KO^Tie2^) mice and *Arg* ^*fl/fl*^ */Tie2-Cre* ^*−/−*^ littermates (Arg1-Con) were analyzed for the presence of the *LysM-Cre* or *Tie2-Cre* transgene by PCR using primer pairs *LysM-F* and *LysM-R* or *Tie2-F* and *Tie2-R*, respectively (Additional file 1: Table S1). The Cre-excised *Arg1* allele (298 bp) was detected with the primers *Arg1-F2* (Additional file 1: Table S1) and *Arg1-R1*. Mice were kept with 2-3 animals per filter-top cage with wood shavings as bedding on a 12-h light/dark cycle, with standard chow and water ad libitum*.* Mice were checked daily for their well being by dedicated personnel of the animal facility.

### Antigen sensitization and challenge

Antigen sensitization and challenge have been described before [33]. In brief, ten-week old female mice were injected intraperitoneally on days 0 and 14 with 10 μg of ovalbumin (OVA), grade V (Sigma-Aldrich, Zwijndrecht, The Netherlands) in the presence of 1 mg/mL of alum adjuvant (Imject Alum®, Thermo Scientific, Rockford, IL, USA). On days 21-27, mice were exposed daily for 30 min to 1% (*w*/*v*) aerosolized OVA in PBS. Lung function was assessed 12 h after the last challenge. Groups, each containing 2-3 Arg1-Con or Arg1-KO mice pretreated with PBS or OVA (in total 7-8 mice per genotype and treatment), were tested at 3-4 different occasions to correct for any “session” effects [37].

### Airway hyperresponsiveness

Assessment of methacholine responsiveness was carried out as previously described [33]. Briefly, 7-8 mice per genotype and treatment were anesthetized with 80 mg/kg sodium pentobarbital and an additional 40 mg/kg 30 min later. An 18-gauge blunt needle was inserted into the trachea and connected to a mechanical small-animal ventilator (FlexiVent, Scireq, Montreal, Canada). Mice were ventilated at 200 breaths/min with a delivered tidal volume of 0.25 mL against a positive end-expiratory pressure (PEEP) of 3 cm H_2_O applied by a water trap. Lungs were challenged by delivering successively 0, 3.1, 12.5 and 50 mg/mL aerosolized methacholine (Sigma) in PBS through the tracheal cannula using an ultrasonic nebulizer during 10 deep inhalations with a tidal volume of 0.8 mL. Following each methacholine challenge, the input impedance (Z_rs_) of the respiratory system was measured. Parameters measured were: R_N_, the Newtonian airflow resistance, mostly in the conducting pulmonary airways; H, “tissue elastance”, or the elastic energy stored in tissues; G, “tissue resistance”, or viscous dissipation of energy in respiratory tissues and airflow heterogeneity.

### Plasma collection and analysis

After each experiment, blood was collected from the inferior caval vein of 7-8 mice per group in heparin-coated tubes, centrifuged 3 min at 5000*g, snap-frozen and stored at −80 °C as described previously [33]. Plasma OVA-specific IgE levels were determined by ELISA (MD Biosciences, M036005, Zürich, Switzerland). For the determination of plasma amino acids, 50 μL of plasma was added to 4 mg sulfosalicylic acid, vortexed, snap-frozen in liquid nitrogen and stored at −80 °C until use. Plasma amino-acid concentrations were measured as described [33].

### Tissue isolation

Following euthanasia, the left lung was filled with 10% buffered formalin (Klinipath, Deventer, The Netherlands) for 10 min at a pressure of 20 cm H_2_O and submersed overnight in 4% formaldehyde at room temperature prior to paraffin embedding. The right lung was snap frozen in liquid nitrogen, pulverized in a liquid-nitrogen-chilled mortar, and stored at −80 °C as described previously [33].

### Immunostaining

Immunostaining was performed as described previously [33]. In brief, paraffin-embedded tissue was cut into 4 μm sections and stained with hematoxylin & eosin (H&E), reticulin, or Sirius red. Epitope retrieval was carried out by heating the slides for 5 min in 10 mM sodium citrate (pH 6 at room temperature (RT)) at 95 °C and cooling to RT for 30 min. Endogenous peroxidase was blocked with peroxidase block (DAKO, S2001, Enschede, the Netherlands) for 10 min at RT. Non-specific antibody binding was blocked with 10% normal goat serum for 30 min. After washing in PBS, slides were incubated with anti-ARG1 (Amsterdam Liver Center, AMS40.11.13), anti-myeloperoxidase (MPO; DAKO), or anti-major basic protein (MBP; kindly provided by Dr. James Lee, MT14.3.7, Mayo Clinic Scottsdale, AZ, USA) as described [33]. After washing, sections were incubated with a 1:200 diluted biotinylated rabbit anti-rat secondary antibody (DAKO) for 45 min at RT, washed, incubated with streptavidin/HRP (Vector) for 30 min at RT, and developed with 3,3′-diaminobenzidine (Sigma) for 10 min. Sections stained for ARG1 were incubated with an AP-labeled secondary antibody (DAKO) for 45 min, developed with nitroblue-tetrazolium and 5-bromo-4-chloro-3-indolyl phosphate (Roche, Almere, The Netherlands) dissolved in 50 mM MgSO4, 100 mM Tris·HCl (pH 9.5) for 30 min, and cover-slipped with an aqueous mounting medium (DAKO). To facilitate quantification, the immunostained slides were not counterstained.

### Histological assessment

Lung inflammation was assessed on H&E-stained sections. MBP and MPO images were digitized with the Pannoramic slide scanner 250 (3DHistech, Budapest, Hungary), and counted using the Pannoramic viewer software application. Color deconvolution was adjusted to detect diaminobenzidine-stained cells. Only cells with a diameter between 10 and 15 μm were included. Arginase-positive cells were counted manually in three different locations (peribronchiolar, perivenous and in the intervening parenchyma). The density of the counted cells was expressed per mm^2^ tissue.

### Milliplex assay

Tissue powder was homogenized in PBS, pH 7.6, in the presence of a proteinase inhibitor cocktail (Complete, Roche). Cytokines (CCL11 (eotaxin-1), IL-4, IL-5, IL-10, IL-13, TNFα, and IFNγ) were quantified using a Luminex ® xMAP® multiplex platform, combined with a customized Milliplex™ mouse chemokine/cytokine panel from Millipore™, as described previously [33].

### RNA isolation and quantification

RNA purification and quantification was performed as described previously [33]. In brief, tissue powder was homogenized in Tri reagent (Sigma) with the Mini Bead-Beater (Biospec products, Bartlesville, OK, USA). To remove genomic DNA, RNA was precipitated with 2 M LiCl for at least 30 min at −20 °C. RNA integrity was checked by denaturing gel electrophoresis. RNA concentration was determined with a NanoDrop-ND-1000 spectrophotometer at 260 nm (Isogen Life Sciences, Wilmington, DE, USA). 400 ng of total RNA was transcribed using a first-strand synthesis kit (Roche). Quantitative PCR was performed in a Lightcycler 480 (Roche), using Lightcycler 480® SYBRgreen mastermix (Roche) and the following settings: denaturation: 30 s at 95 °C; annealing 30 s at 60 °C; elongation 30 s at 72 °C; 45 cycles; and a final elongation step for 5 min at 72 °C. If reverse transcriptase was omitted, no product was formed. Primary fluorescence data were quantified as described [33] and expressed relative to that of *18S* rRNA. Primer sequences are given in Additional file 1: Table S1.

### Western blot

Western blot analysis was performed as described previously [33]. In brief, tissue powder was homogenized in RIPA buffer (25 mM Tris·HCl (pH 7.6), 150 mM NaCl, 1% NP-40, 1% Na-deoxycholate, 0.1% SDS, containing Complete® cocktail (Roche). Protein concentration was measured with the bicinchoninic-acid assay (Pierce, Rockford, IL, USA). Twenty-five μg protein was separated by SDS-PAGE, transferred onto 0.45 μm nitrocellulose membranes, using a wet transfer system (Biorad, Hercules, CA, USA). Equal loading of lanes was confirmed by Ponceau S staining, followed by a wash-step with TBS (50 mM Tris, 150 mM NaCl, pH 7.6) and blocking of non-specific binding with 5% skimmed milk in TBS/0.5% Tween-2. Rabbit anti-ARG1 antibody (1:200), followed by an HRP-conjugated swine anti-rabbit secondary antibody (DAKO) was used to visualize ARG1. Signal was developed with the Super Signal West Pico Substrate (Pierce) and quantified in a Fuji systems darkbox (Fuji Film Life Sciences, Tokyo, Japan).

### Statistical analyses

Groups were compared using the Kruskal-Wallis test for PBS/OVA- versus OVA/OVA-treated, and Arg1-Con versus Arg1-KO^Tie2^ mice, as described previously [33]. If this nonparametric test indicated a difference, a multiple comparison of the groups was carried out. Values were considered as statistically significant if *P* < 0.05, and as indicating a trend if *P* < 0.10.

The bivariate two-tailed Pearson correlation coefficients between each of the lung-function parameters, mRNA and protein concentrations, histology quantification data and plasma amino-acid concentrations were determined after combining the data from the comparable PBS/OVA and OVA/OVA groups. In the Tables, *P*-values of the resulting correlation coefficients were color-coded, with red indicating a *P* < 0.001, orange 0.01 > *P* > 0.001, and yellow 0.05 > *P* > 0.01.

## Results

### *Arg1* expression in the lungs of OVA-sensitized and -challenged mice with *Arg1*-deficient macrophages

**Fig. 1:**
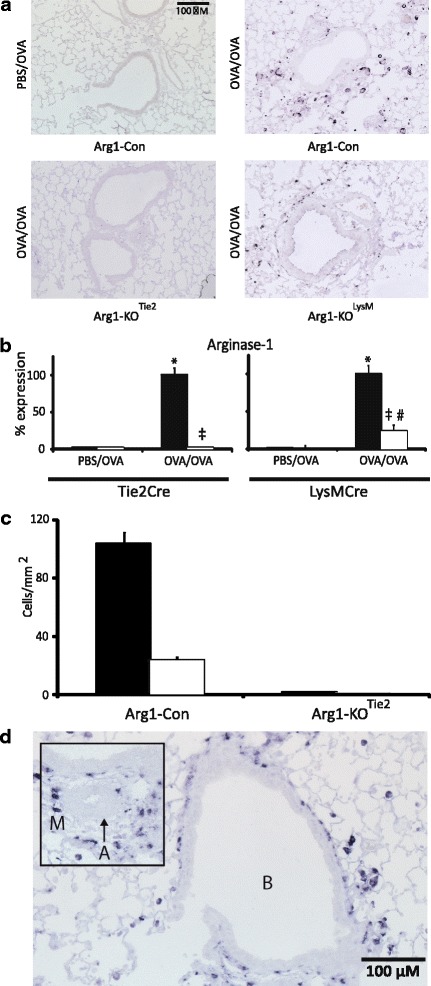
Validation of *Arg1* ablation in lung of female *Arg1* ^*fl/fl*^ mice. **a** Lungs of PBS/OVA- and OVA/OVA-treated female control mice, respectively (top row), and of OVA/OVA-treated female Arg1-KO^Tie2^ and Arg1-KO^LysM^ mice, respectively (bottom row). Note the complete absence of ARG1-positive macrophages in Arg1-KO^Tie2^ mice and the reduction in Arg1-KO^LysM^ mice. **b** Reduction of ARG1 protein content in the lungs of OVA/OVA-treated Arg1-KO^Tie2^ and Arg1-KO^LysM^ mice (white bars) compared to the corresponding Arg1-Con^LysM^ or Arg1-Con^Tie2^ mice (black bars). Means ± SEM (*n* = 8 mice per group). **c** Number of ARG1-positive cells per mm^2^ lung tissue. Cells were counted near bronchioles and arteries (black bars) and in the lung parenchyma (white bars). Means ± SEM are shown (*n* = 8 mice per group). **d** Detail of ARG1 protein content in OVA/OVA-treated control lung. The absence of ARG1 in arterial endothelium is noteworthy. Abbreviations: A = artery, and M = ARG1-positive macrophages. Significance symbols: *: *P* < 0.001 OVA/OVA vs. PBS/OVA Arg1-Con; #: *P* < 0.001 OVA/OVA vs. PBS/OVA Arg1-KO^Lysm^; ‡: *P* < 0.001 OVA/OVA Arg1-KO^Lysm^ or Arg1-KO^Tie2^ vs. OVA/OVA Arg1-Con

**Fig. 2:**
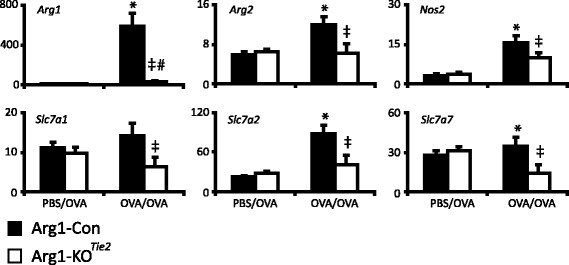
Effect of *Arg1* ablation in the lung on the expression of arginine-metabolizing enzymes and arginine transporters. Black bars represent Arg1-Con mice and white bars Arg1-KO^Tie2^ mice. The specific treatment (PBS/OVA or OVA/OVA) is indicated below the columns. mRNA abundance (AU = number of mRNA copies normalized to 18S rRNA expression and multiplied by 10^4^) of the arginine-metabolizing enzymes *Arg1, Arg2,* and *Nos2* and the arginine transporters *Slc7a1, Slc7a2* and *Slc7a7* is shown as mean ± SEM of 7-8 mice per group. Significance symbols: *: *P* < 0.05 OVA/OVA vs. PBS/OVA Arg1-Con; #: *P* < 0.05 OVA/OVA vs. PBS/OVA Arg1-KO^Tie2^; ‡: *P* < 0.05 OVA/OVA Arg1-Con vs. Arg1-KO^Tie2^

Arg1-KO^LysM^ mice, Arg1- KO^Tie2^ mice, and their Arg1-Con littermates were either sensitized-and-challenged with ovalbumin (OVA/OVA), or mock-sensitized and then challenged with ovalbumin (PBS/OVA). As expected, we found no ARG1-positive cells in the lungs of PBS/OVA-treated mice, whereas the lungs of OVA/OVA-treated Arg1-Con mice contained many ARG1-positive cells (Fig. 1a). ARG1-positive cells were completely absent from the lungs of OVA/OVA-treated Arg1-KO^Tie2^, whereas the number of ARG1-positive cells was reduced, but not eliminated, in the lungs of similarly treated Arg1-KO^LysM^ mice. Of note, we observed no appreciable ARG1 in the endothelial cells in PBS/OVA (Fig. 1a) or OVA/OVA-treated lung blood vessels (Fig. 1d). Furthermore, we did not observe any ARG1 protein or mRNA expression in the lungs of mice undergoing the PBS/OVA protocol, whereas a robust induction of ARG1 protein and mRNA expression was found in the lungs of Arg1-Con mice exposed to the OVA/OVA protocol (black columns in Figs. 1b and 2). Pulmonary *Arg1* mRNA and protein concentration in OVA/OVA-treated Arg1-KO^Tie2^ mice were reduced to 5 ± 2% and 2 ± 0.2%, respectively, and in OVA/OVA-treated Arg1-KO^LysM^ mice to 20 ± 8% and 19 ± 10%, respectively, of that found in similarly treated Arg1-Con littermates (Figs. 1b and 2, white columns). We also counted ARG1-positive cells surrounding bronchioles and arteries, and in parenchyma of OVA/OVA-treated Arg1-Con and Arg1-KO ^Tie2^ mice, and found no ARG1-positive cells in lung tissue of Arg1-KO ^Tie2^ mice (Fig. 1c). From this finding, we conclude that the floxed 4^th^ exon of *Arg1* was accessible to the Cre enzyme and that the reduction in *Arg1* expression was near complete in female Arg1-KO^Tie2^ mice (reduction to ~2%), whereas residual *Arg1* expression was present in female Arg1-KO^LysM^ mice (reduction to ~20%). Since Arg1-KO^LysM^ mice showed an incomplete elimination of *Arg1* expression and an intermediate phenotype, we limit the description of our study to Arg1-KO^Tie2^ mice and their Arg1-Con littermates.

### Effect of *Arg1* deletion on arginine-metabolizing and -transporting genes in the lung

We investigated to what extent ablation of *Arg1* in the lung caused changes in the expression of arginine-metabolizing enzymes and arginine transporters. Figure 2 shows that the pulmonary mRNA abundance of *Arg1, Arg2, Nos2, Slc7a1, Slc7a2* and *Slc7a7* was induced by the OVA/OVA protocol compared to the PBS/OVA protocol. More importantly, the induction was consistently lower in Arg1-KO^Tie2^ than in Arg1-Con female mice subjected to the OVA/OVA protocol. As a result, OVA/OVA treatment no longer induced the expression of *Arg2*, *Nos2*, *Slc7a1, Slc7a2*, and *Slc7a7* in Arg1-KO^Tie2^ mice. These data clearly show that effective ablation of *Arg1* expression in macrophages suppressed the induction of arginine-metabolizing enzymes and arginine transporters in the lungs of OVA/OVA-treated female mice.

### The effect of *Arg1* ablation on circulating amino acids

OVA/OVA treatment of female Arg1-Con mice caused a significant drop in the concentration of circulating arginine levels relative to their PBS/OVA-treated littermates (Additional file 1: Table S2). The decline was not seen in OVA/OVA-treated Arg1-KO^Tie2^ mice, implying an effect of ARG1. In both Arg1-KO^Tie2^ and Arg1-Con mice, OVA/OVA treatment also caused an increase in the plasma concentration of ornithine and a decline in that of glycine relative to the corresponding PBS/OVA-treated mice (Additional file 1: Table S2). As a result, the arginine bioavailability index (the ratio of arginine and ornithine *plus* lysine concentrations) decreased non-significantly to ~71% (*P* = 0.369) in OVA/OVA-treated Arg-Con, but remained unchanged in Arg1-KO^Tie2^ mice.

### Ablation of *Arg1* in the lung has no effect on allergen-induced airway hyperresponsiveness

**Fig. 3:**
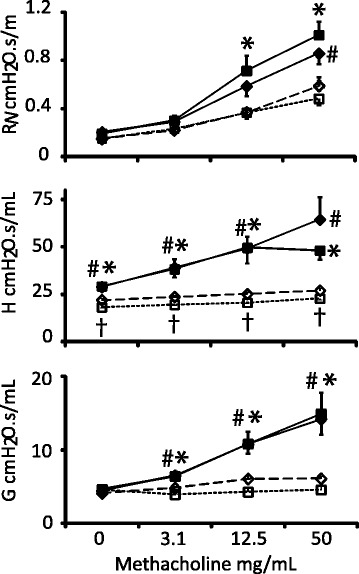
Effect of *Arg1* ablation in the lung on lung function as measured with the FlexiVent. PBS/OVA-treated Arg1-Con mice are represented by open diamonds connected by dashed lines and PBS/OVA-treated Arg1-KO^Tie2^ mice by open squares connected by dotted lines. OVA/OVA-treated Arg1-Con mice are represented by filled diamonds and OVA/OVA treated Arg1-KO^Tie2^ mice by filled squares both connected by continuous lines. Airway resistance R_N_, tissue elastance H, and tissue resistance G are shown in panels A, B, and C, respectively. Values on the X-axis indicate the concentrations of aerosolized methacholine. Means ± SEM (*n* = 7-8 mice per group). Significance symbols: #: *P* < 0.01 (OVA/OVA vs. PBS/OVA Arg1-Con); *: *P* < 0.01 (OVA/OVA Arg1-KO^Tie2^ vs. PBS/OVA Arg1-KO^Tie2^); †: *P* < 0.01 (PBS/OVA Arg1-KO^Tie2^ vs. PBS/OVA Arg1-Con). Arg1-KO^Tie2^ and Arg1-Con mice did not differ in their response to OVA/OVA treatment

To address the question whether *Arg1* ablation in the lung affects respiratory mechanics, we measured methacholine responsiveness in PBS/OVA- and OVA/OVA-treated Arg1-KO^Tie2^ mice and their Arg1-Con littermates (Fig. 3). Compared to PBS/OVA treatment, OVA/OVA treatment increased airway resistance (R_N_), tissue elastance (H), and tissue resistance (G) in female Arg1-Con and Arg1-KO^Tie2^ mice, without an effect of *Arg1* depletion. Collectively, these findings show that *Arg1* ablation in the lung did not affect AHR in female mice.

### Ablation of *Arg1* in the lung affects mRNA abundance of inflammatory genes

**Fig. 4:**
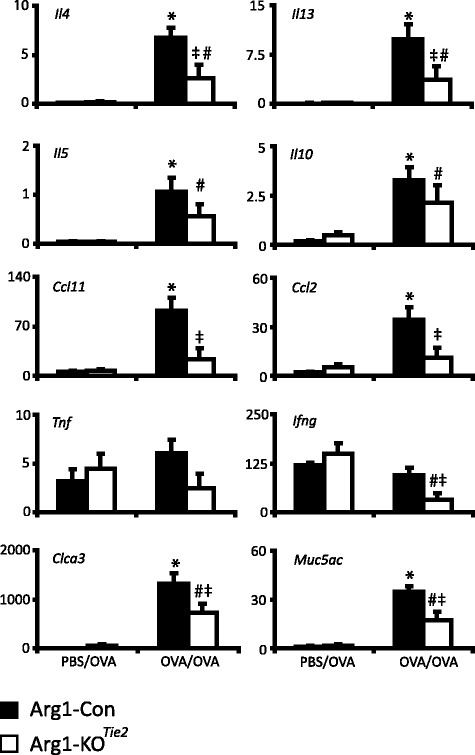
Effect of *Arg1* ablation in the lung on the expression of inflammatory genes. Black bars represent Arg1-Con mice and white bars Arg1-KO^Tie2^ mice. mRNA abundance (AU = number of mRNA copies normalized to 18S rRNA expression and multiplied by 10^4^) of the T_H_2-related inflammatory genes *Il4, Il13, Il5* and *Ccl11*, the macrophage-chemotactic protein *Ccl2,* the anti-inflammatory gene *Il10,* the T_H_1-related inflammatory genes *Tnfa* and *Ifng,* and the marker genes for the activation of bronchiolar epithelium *Muc5ac* and *Clca3* are shown as means ± SEM (*n* = 7-8 mice per group). Significance symbols: *: *P* < 0.05 (OVA/OVA vs. PBS/OVA Arg1-Con); #: *P* < 0.05 (OVA/OVA vs. PBS/OVA Arg1-KO^Tie2^); ‡: *P* < 0.05 (OVA/OVA Arg1-KO^Tie2^ vs OVA/OVA Arg1-Con)

We investigated whether *Arg1* ablation in the lung affected the expression of asthma-associated cytokines (Fig. 4). OVA/OVA treatment increased the abundance of *Il13, Il4, Il5, Ccl2, Ccl11 (Eotaxin-1),* and *Il10* mRNAs in Arg1-Con mice as expected, while the expression of *Tnfa* and *Ifng* remained unchanged. Furthermore, OVA/OVA treatment of Arg1-Con mice increased gene expression of respiratory epithelium-specific *Muc5ac* and *Clca3*. OVA/OVA-treated female Arg1-KO^Tie2^ mice differed from their OVA/OVA-treated Arg1-Con littermates by showing a significantly lower expression of the *Il13, Il4*, *Ccl2, Ccl11*, *Ifng, Muc5ac*, and *Clca3* mRNAs. These findings imply a direct relation between *Arg1* elimination in the lung and the decreased expression of inflammatory genes in the lungs of OVA/OVA-treated mice.

### *Arg1* ablation in the lung does not affect protein levels of cytokines and chemokines

**Fig. 5:**
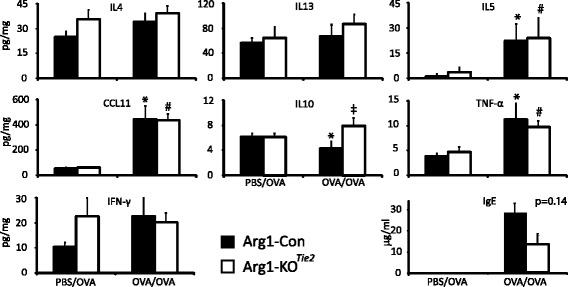
Effect of *Arg1* ablation in the lung on the pulmonary concentration of inflammatory cytokines, chemokines, and OVA-specific IgE. Black bars represent Arg1-Con mice and white bars Arg1-KO^Tie2^ mice. Treatment of the mice (PBS/OVA or OVA/OVA) is indicated. The pulmonary concentrations of IL-4, IL-13, IL-5, CCL11, IL-10, TNFα and IFNγ (pg/mg total protein), and OVA-specific IgE (ng/mL) in plasma are shown as means ± SEM (*n* = 7-8 mice per group). Significance symbols: *: *P* < 0.05 (OVA/OVA vs. PBS/OVA Arg1-Con); #: *P* < 0.05 (OVA/OVA vs. PBS/OVA Arg1-KO^Tie2^); ‡: *P* < 0.05 (OVA/OVA Arg1-KO^Tie2^ vs OVA/OVA Arg1-Con)

To determine whether *Arg1* ablation in the lung had an effect on the protein concentration of pulmonary cytokines and chemokines, we measured IL-4, IL-5, IL-10, IL-13, CCL11, TNFα and IFNγ protein in extracts of whole-lung homogenates (Fig. 5). Compared to PBS/OVA-treated female mice, OVA/OVA-treated Arg1-Con and Arg1-KO^Tie2^ mice had increased concentrations of IL-5, CCL11 and TNFα in their lungs, whereas the concentration of IL-4, IL-13, and IFNγ was unchanged. IL-10 was lower in OVA/OVA- than in PBS/OVA-treated Arg1-Con mice and higher in OVA/OVA- treated Arg1-KO^Tie2^ mice than in similarly treated Arg1-Con mice. Apart from IL-10, no differences in cytokine and chemokine protein concentration were found between OVA/OVA-treated Arg1-Con and Arg1-KO^Tie2^ mice.

### Effect of *Arg1* deletion on the immune response to OVA

We investigated whether the production of ovalbumin-specific IgE antibodies was affected by the ablation of *Arg1*. OVA-specific IgE was not detectable in plasma of PBS/OVA-treated mice. OVA-specific IgE levels in OVA/OVA-treated female Arg1-KO^Tie2^ mice were reduced to ~50% of that in OVA/OVA-treated Arg1-Con mice, but due to the high variance in plasma IgE concentrations this difference did not reach statistical significance (*P* = 0.14).

### Effects of *Arg1* deletion in the lung on pulmonary inflammation

**Fig. 6:**
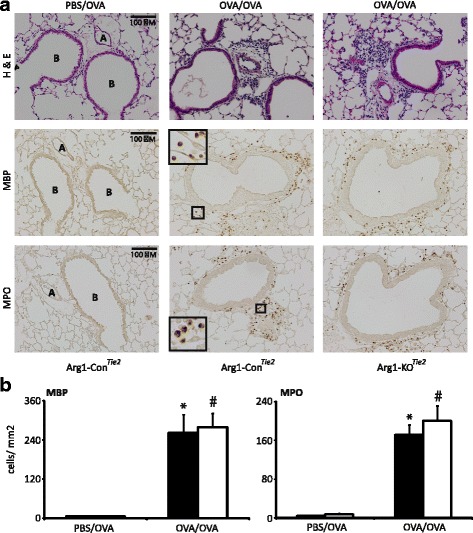
Effect of *Arg1* ablation in the lung on the prevalence of inflammatory cells in the lungs. A: The left two columns show lungs of PBS/OVA- and OVA/OVA-treated Arg1-Con mice, while the right column shows lungs of OVA/OVA-treated Arg1-KO^Tie2^ mice. Top row: hematoxylin and eosin; middle row: MBP staining for eosinophils; bottom row: MPO staining for neutrophils. Bar: 100 μm. B: Quantification of the inflammatory cells in OVA/OVA-treated Arg1-Con (black bars) and Arg1-KO^Tie2^ (white bars) mice. All sections were stained simultaneously. Means and SEM of 7-8 mice per group are shown. *: *P* < 0.05 (OVA/OVA vs. PBS/OVA Arg1-Con); #: *P* < 0.05 (OVA/OVA vs. PBS/OVA Arg1-KO^Tie2^)

**Fig. 7:**
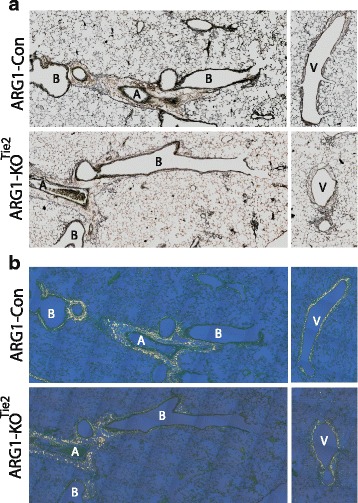
Effect of *Arg1* ablation on lung remodeling. **a**: Bright-field images of reticulin-stained sections of OVA/OVA-treated female Arg1-Con and Arg1-KO^Tie2^ mice. **b**: Polarized-light images of Sirius red-stained sections of OVA/OVA-treated female Arg1-Con and Arg1-KO^Tie2^ mice to assess collagen content (serial sections of panel A). The left-sided panels show sections of periarterial areas and the right-handed panels sections of perivenous areas. Abbreviations: B = bronchiole, A = artery, and V = vein

We investigated the effect of *Arg1* ablation on the OVA/OVA-induced inflammatory response in lung tissue (Fig. 6). H&E-stained sections of lung tissue did not reveal any inflammatory cells in PBS/OVA-treated mice (Fig. 6A). This finding was confirmed by staining the same lungs for the presence of eosinophils and neutrophils using MBP and MPO as markers, respectively (Fig. 6A). As expected, many inflammatory cells were present in the lungs of OVA/OVA-treated mice, but their number was not different between female Arg1-KO^Tie2^ mice and their Arg1-Con littermates (Fig. 6B). To investigate whether there was a difference in lung remodeling between female Arg1-KO^Tie2^ mice and their Arg1-Con littermates, we stained slides for reticulin and collagen (Sirius red), but did not detect differences either (Fig. 7).

### Comparison of responses to allergic asthma in female Arg1-Con and Arg1-KO^Tie2^ mice

**Fig. 8:**
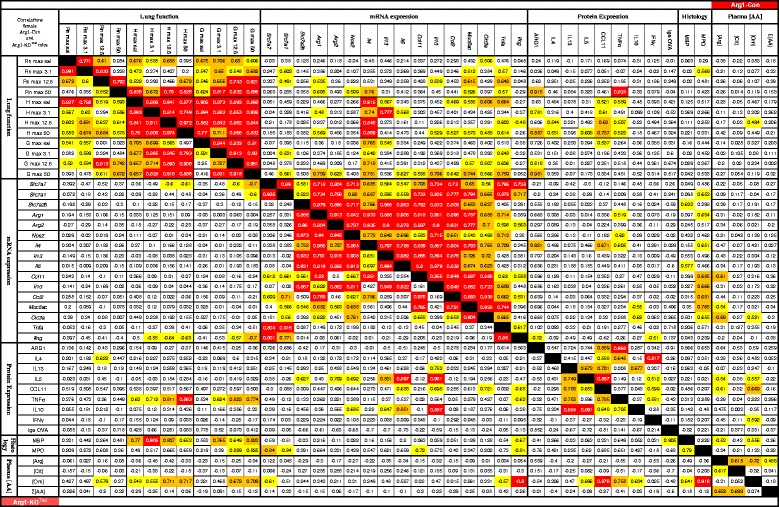
Effect of *Arg1* ablation in the lung on the correlation of lung function, abundance of pulmonary mRNAs and proteins, pulmonary histopathology, and concentrations of plasma amino acids in female mice. The triangle on the upper right side refers to data from Arg1-Con mice and the triangle on the lower left to data from Arg1-KO^Tie2^ mice. The correlation coefficients between the parameters named above the columns and left to the rows, respectively, as measured in all 15 or 16 PBS/OVA- and OVA/OVA-treated mice of the Arg1-Con or Arg1-KO^Tie2^ groups are shown. The significance of the correlations is color-coded: yellow: 0.05 > *P* > 0.01, orange: 0.01 > *P* > 0.001, and red: *P* < 0.001. The near-absence of cross-correlations of parameters between the categories named in the title and the decline of the number or degree of significant correlations in Arg1-KO^Tie2^ compared to Arg1-Con mice is noteworthy

To investigate whether the presence or absence of ARG1 activity affected the adaptive responses to OVA-induced asthma in a quantitatively similar way, we also investigated to what extent changes in lung-function parameters, mRNA and protein concentrations, histology quantitation and plasma amino-acid concentrations correlated in either Arg1-Con or Arg1-KO^Tie2^ mice (Fig. 8). Strikingly, lung-function parameters and mRNA concentrations in Arg1-Con females strongly corresponded within, but hardly between both categories. Noticeable exceptions were a correlation between the response of lung-function parameters H and G and the expression of *Il4* mRNA, and the near absence of a correlation between the response of the pro-inflammatory cytokines *Tnfa* and especially *Ifng*, and all other mRNAs measured. Female Arg1-KO^Tie2^ differed from Arg1-Con mice (Fig. 8) by a pronounced decrease in the tightness of the correspondence between the adaptive responses that were mounted. Striking examples were the loss of correspondence between the response of H and G, and *Il4* mRNA expression, and the loss of correspondence of the expression of the mRNAs of arginine transporters *Slc7a7* and *Slc7a1*, and that of all other mRNAs.

## Discussion

*Tie2Cre*-dependent deletion of *Arg1* expression in myeloid cells prevented the expression of ARG1 in lung inflammatory cells of OVA/OVA-treated Arg1-KO^Tie2^ mice. Compared to female OVA/OVA-treated Arg1-Con mice, Arg1-KO^Tie2^ mice had lower OVA-specific IgE levels and a lower expression of arginine-metabolizing or -transporting genes (*Arg2, Nos2, Slc7a1, Slc7a2,* and *Slc7a7*), of cyto- and chemokine genes (*Il4, Il13, Il5, Ccl11, Ccl2,* and *Ifng*), and of epithelial marker genes (*Muc5ac* and *Clc3a*), but *Arg1* deficiency did not affect airway resistance (R_N_), tissue elastance (H), or tissue resistance (G). Furthermore, the tightness of the correspondence between the adaptive responses that were mounted in OVA/OVA-treated female mice were strongly reduced if these mice were unable to express *Arg1* in their alveolar macrophages.

### Efficacy of *Tie2Cre*-mediated *Arg1* ablation in allergically inflamed lungs

*Tie2Cre* mice excise *loxP*-flanked DNA in both endothelial and early hematopoietic progenitor cells [36, 38]. As in male mice [33] and in other studies [34, 36, 39, 40], *Tie2Cre*-mediated excision of *Arg1* resulted in a near-complete deletion of *Arg1* mRNA and protein expression in macrophages. A near-complete *Arg1* excision was also observed in peritoneal macrophages of Arg1-KO^Tie2^ mice [41]. Importantly, we did not observe ARG1 protein in the endothelium of the pulmonary blood vessels of PBS/OVA- or OVA/OVA-treated Arg1-Con mice (Fig. 1a,d), even after prolonged incubation with the alkaline-phosphatase substrate (up to 90 min). In support of this finding, most other studies found no or only minimal arginase1 protein in lung epithelium and smooth muscle [42–44], also after inducing allergic inflammation [45–47]. Based on these findings, we conclude that Arg1-KO^Tie2^ mice can be used for the ablation of *Arg1* in macrophages of allergically inflamed lungs.

The near 100% efficacy of *Tie2Cre*-mediated excision of *loxP*-flanked *Arg1* ^*fl*^ sequences contrasts with the 70-80% efficacy *LysMCre*-mediated *Arg1* ^*fl*^ excision found in the present study and in our earlier study in male Arg1-KO mice [33]. Other studies [35, 48–50] also report *LysMCre*-mediated target excision in only 50-80% of the target cells. Since the 20% remaining expression of *Arg1* in the lungs of OVA/OVA-treated Arg1-KO^LysM^ mediated an intermediate phenotype between OVA/OVA-treated Arg1-Con and Arg1-KO^Tie2Cre^ mice, we limited our description of the effects of pulmonary *Arg1* deficiency on allergic asthma to Arg1-KO^Tie2Cre^ mice.

### *Arg1* ablation in macrophages and arginine availability do not affect lung biomechanics in allergically inflamed mouse lungs

Lung mechanics were measured using the forced-oscillation technique, which yields data on airway resistance (R_N_) in the large airways, and on tissue elastance (H) and resistance (G) that reflect the function of the peripheral parts of the lung [51, 52]. Arg1-KO^Tie2^ and Arg1-Con mice did not differ in any of these parameters with or without OVA/OVA treatment. This finding in female mice contrasts with our earlier observation in male mice, in which *Arg1* deficiency in macrophages improved the peripheral lung mechanics parameters H and G [33]. Since the experiments in male and female mice were carried out concurrently with mice from the same litters, the difference cannot be attributed to a session effect. However, the present finding does agree with studies in which deficiency of *Arg1* in macrophages was mediated by bone-marrow transfer of constitutively *Arg1*-deficient stem cells [53] or *Tie2Cre*-mediated elimination of *Arg1* exons 7 and 8 [34]. Both studies tested, in addition to the OVA/OVA protocol, allergic inflammation in response to *Aspergillus fumigatus* and *Schistosoma mansoni* eggs in both C57BL/6 and BALB/c mice, but did not state whether the lung function tests were carried out in male and/or female mice.

The lack of an effect of *Arg1* deficiency on lung mechanics is in line with a smaller increase in the expression of arginine-metabolizing and -transporting genes upon OVA/OVA treatment (Fig. 2). Administration of pharmacological arginase inhibitors reduced AHR of allergen-induced asthma in both male and female mice. Whereas arginase inhibitors reduced lung inflammation in male mice [28, 29], they enhanced inflammation in female mice [26]. Because we observed no effect on AHR and a reduced inflammatory response in *Arg1*-deficient lungs, the effects of systemic inhibition of arginase-1 and -2 activities apparently differ from those of a deletion of *Arg1* from macrophages. Arginine analogues may exert additional effects, as NOS2 depletion or inhibition did not affect AHR, but that of NOS1- and −3 did [30, 54, 55].

The circulating concentration of L-arginine (Additional file 1: Table S2) was ~17% lower in OVA/OVA-treated Arg1-Con mice than in similarly treated Arg1-KO^Tie2^ mice, showing that *Arg1* expression in hematopoietic tissue had an impact on plasma arginine. Because the concentration of plasma ornithine increased and that of citrulline was unchanged, the plasma [Arg]/([Orn] + [Cit]) index decreased, in agreement with a recent report in female BALB/c mice [56]. Scott et al. [56] attributed an important role to lung arginase in the increase of plasma ornithine, but we observed a similar increase in plasma ornithine in mice with and without arginase1 in their lungs. Furthermore, we did not find an increase in plasma ornithine or a decrease in the arginine availability index in OVA/OVA-treated male Arg1-Con and Arg1-KO mice [33], even though methacholine responsiveness was decreased by *Arg1* deletion in male mice. The plasma [Arg]/([Orn] + [Lys]) index, which may reflect the availability of basic amino acids, only declined in OVA/OVA-treated female Arg1-KO mice. The question, therefore, is whether plasma arginine concentration or any plasma arginine availability index is important for respiratory function. Barron et al. [34] suggested that the enormous perfusion of the lung, which equals the cardiac output, assures that the supply of arginine from plasma to tissue cells is hardly impaired by local arginase activity. In addition, we have recently shown that citrulline rather than arginine is the more efficient extracellular source for intracellular arginine [57].

### *Arg1* ablation does not affect pro-inflammatory protein content in allergically inflamed lungs

OVA/OVA treatment of Arg1-KO mice resulted in a similar degree of lung infiltration with inflammatory cells as seen in Arg1-Con littermates, but *Il13*, *Il4*, *Il5, Ccl2, Ccl11, Ifng, Muc5ac,* and *Clca3* were expressed to a lesser extent in Arg1-KO ^Tie2^ than in Arg1-Con mice. Such a coordinated transcriptional response of genes probably reflects the activity of one or more common regulatory genes or proteins. However, as far as measured, changes in mRNA abundance of the chemo- and cytokines were not reflected in the corresponding protein concentrations in lung tissue (Fig. 8). This finding can be explained by the fact that cyto- and chemokines are secreted proteins, but inefficient translation of the mRNAs in Arg1-KO mice can also contribute, because posttranscriptional regulation of expression is a well-known feature of cyto- and chemokines [58]. In OVA/OVA-treated Arg1-Con males, we did observe a reasonable correspondence between mRNA and protein levels in 4 out of 8 combinations measured, but this correspondence disappeared in OVA/OVA-treated Arg1-KO male mice. The decline in circulating arginine concentration in OVA/OVA-treated males [33] was similar to that observed in females. Low circulating arginine concentrations are known to affect translation by inducing endoplasmic reticulum (ER) stress [59].

### Comparison of the adaptive responses to OVA sensitization and challenge in male and female Arg1-Con mice

In a parallel study, we investigated the effects of *Arg1* ablation in the lung of genetically identical, similarly treated male mice [33]. Since the biology of female mice is underreported and since females are more often affected by allergic asthma than males [4–6, 60], we compared the adaptive responses of Arg1-Con mice in both sexes (Additional file 1: Table S3). The OVA-specific IgE concentration was ~3-fold higher in plasma of OVA/OVA-treated female than male mice. The sex difference in IgE accumulation is well-established [4]. *Arg1* deficiency did not reduce the induction of plasma IgE concentration in males, but tended to reduce it by ~2-fold in females. However, this reduction in plasma IgE did not modify the inflammatory response in OVA/OVA-treated *Arg1*-deficient lungs. OVA/OVA-treated BALB/c female mice further have higher lung concentrations of IL-4, IL-5, IL-10, IL-13, IFN-γ, and TNF-α protein [4, 6, 61] than male BALB/c mice, but we observed similar IL-4, IL-13, IL10, CCL11, TNFα, and IFNγ concentrations in OVA/OVA-treated male and female C57BL/6 mice. It is well-recognized that C57BL/6 and BALB/C mice differ in their response to OVA/OVA treatment [62, 63].

OVA/OVA-treated Arg1-Con females accumulated ~3-fold more *Arg1* mRNA than similarly treated males, without showing different numbers of arginase1-positive macrophages in the lung. As far as we are aware, a sex difference in macrophage *Arg1* mRNA and protein expression in OVA/OVA-treated mice has not yet been reported. Furthermore, the expression of *Il4*, *Il13* and, to a lesser extent, *Il5* was affected by *Arg1* deletion in female mice only, suggesting a sex difference in the expression of M2 cytokines in OVA/OVA-treated mice. In Coxsackie virus-induced myocarditis, the ~4-fold higher *Arg1* expression in macrophages of female over male mice was attributed to skewing of the macrophage polarization towards an M2 phenotype in females, and towards an M1 phenotype in males [64]. Depending on the inducing agent, alveolar macrophages can also develop an M1, M2, or even an intermediate phenotype [13]. These sex difference may arise because female steroid hormones promote alternative activation of macrophages, whereas testosterone inhibits the M2 phenotype [65, 66].

We also studied whether there were sex differences in the coordination of the responses to OVA/OVA treatment (Additional file 2: Figure S1). Adaptive changes in methacholine responsiveness and mRNA expression (except the M1-macrophage markers *Ifng* and, to a lesser extent, *Tnfa*) correlated more strongly within their category in females than in males, whereas, as already discussed, IL-4, IL-13, IL-5, CCL11, and TNFα protein concentrations correlated better with the corresponding mRNA concentrations and with lung function parameters H and G in males. These data imply that the respiratory responses to OVA/OVA treatment were less integrated with the inflammatory responses in female than in male mice.

## Conclusion

Complete ablation of *Arg1* in the lung does not affect methacholine responsiveness or the invasion of inflammatory cells, but does change the gene-expression profile of these cells in OVA/OVA-treated female mice. Since the asthmatic responses in mice and humans show similar sex-related differences, and since ablation of *Arg1* did improve peripheral lung function in male mice only, we hypothesize that treatment of asthma by modulating arginase activity in the lung will primarily benefit male patients.

## Additional files

Additional file 1: Tables S1-S3. **Table S1.** Primers pairs used in genotyping and quantitative PCR. **Table S2.** Amino-acid concentrations in venous plasma (μM ± SEM) of Arg1-Con and Arg1-KO^Tie2Cre^ mice *: *P* < 0.05 OVA/OVA vs. PBS/OVA Arg1-Con; #: *P* < 0.05 OVA/OVA vs. PBS/OVA Arg1-KO^Tie2^. **Table S3.** Differences between male and female Arg1-Con mice after treatment with the OVA/OVA protocol. (DOCX 24 kb)

Additional file 2: Figure S1. Correlation of lung-function, abundance of pulmonary mRNAs and proteins, pulmonary histopathology, and concentrations of plasma amino acids in wild-type male and female mice. The lower-left triangle refers to data obtained female and the upper-right triangle to data from male Arg1-Con mice. The numbers show the correlation coefficients between parameters indicated above and to the left of the columns and rows, respectively, as measured in all 15 or 16 male and female Arg1-Con mice studied, that is, both the PBS/OVA- and the OVA/OVA-treated groups. The significance of the correlations is color-coded according to the *P*-value of the correlation coefficient: yellow: 0.05 > *P* > 0.01, orange: 0.01 > *P* > 0.001, and red: *P* < 0.001. Note the difference in the degree of cross-correlations of parameters between the categories named in the title in males and females. (PDF 219 kb)

## Abbreviations

AHR: airway hyperresponsiveness
ARG1, *Arg1*: arginase 1 protein or *gene*
Arg1-Con: *Arg* ^*fl/fl*^ */LysM-Cre* ^*−/−*^ or *Arg* ^*fl/fl*^ */Tie2-Cre* ^*−/−*^ control littermates
Arg1-KO^LysM^ or Arg1-KO^Tie2^: *Arg* ^*fl/fl*^ */LysM-Cre* ^*tg/−*^ or *Arg* ^*fl/fl*^ */Tie2-Cre* ^*tg/−*^ tissue-specific knockout mice
*Arg2*: arginase 2 gene
*Cat2*: cationic amino acid transporter aka *Slc7a2*
*Ccl11*: C-C motif chemokine 11, aka *eotaxin-1*
*Ccl2*: C-C-motif chemokine ligand 2
*Clca3*: chloride channel accessory 3
G: tissue resistance
H: tissue elastance
H&E: hematoxylin & eosin
IFNγ, *Ifng*: interferon gamma
IgE: immunoglobulin E
IL10: interleukin 10
IL13: interleukin 13
IL4: interleukin 4
IL5: interleukin 5
MBP: major basic protein
MPO: myeloperoxidase
*Muc5ac*: mucin 5 ac
NOS2: nitric oxide synthase
OVA: ovalbumin
OVA/OVA: ovalbumin-sensitized and challenged
PBS/OVA: mock-sensitized and ovalbumin-challenged
R_N_: Newtonian airflow resistance
RT: room temperature
*Slc7a1*: solute carrier family 7 member 1, aka as *Cat1*
*Slc7a2*: solute carrier family 7 member 2, aka as *Cat2*
*Slc7a7*: solute carrier family 7 member 7
TNFα, *Tfna*: tumour necrosis factor alpha
