# Supplementary material for: Arginase 1 deletion in myeloid cells affects the inflammatory response in allergic asthma, but not lung mechanics, in female mice
Source: BMC Pulm Med. 2017 Nov 28;17:158. doi: 10.1186/s12890-017-0490-7 (PMC5706166; doi:10.1186/s12890-017-0490-7)
Supplement: Supplementary file 1 — Table S1. Primers pairs used in genotyping and quantitative PCR. Table S2. Amino-acid concentrations in venous plasma (μM ± SEM) of Arg1-Con and Arg1-KOTie2Cre mice *: P < 0.05 OVA/OVA vs. PBS/OVA Arg1-Con; #: P < 0.05 OVA/OVA vs. PBS/OVA Arg1-KOTie2. Table S3. Differences between male and female Arg1-Con mice after treatment with the OVA/OVA protocol. (DOCX 24 kb) [file 12890_2017_490_MOESM1_ESM.docx]

**Arginase 1 deletion in myeloid cells affects the inflammatory response in allergic asthma, but not lung mechanics in female mice**

Roy H.E. Cloots^1^, Selvakumari Sankaranarayanan^1^, Chiel de Theije^1^, Matthew H. Poynter^3^, Els Terwindt^1^, Wouter H. Lamers^1,2^, and S. Eleonore Köhler^1^

Online data supplement

**Supplemental Table 1. Primers pairs used in genotyping and quantitative PCR**

| Gene | Primer name | Primer sequence (5’-3’) |
| --- | --- | --- |
| Excised *arginase-1* | Arg1-F2 | TCTAGAACTAGTGGATCACCTCAG |
|  | Arg1-R1 | GTGCCTTGGTCTACATTGAACATAC |
| *Cre* | CRE-F | GGTTCGCAAGAACCTGATGGACAT |
|  | CRE-R | GCTAGAGCCTGTTTTGCACGTTCA |
| *Tie-2-Cre* | Tie2-F | CGCATAACCAGTGAAACAGCATTGC |
|  | Tie2-R | CCCTGTGCTCAGACAGAAATGAGA |
| *LysMCre* | LysM-F | GGT TCG CAA GAA CCT GAT GGA CAT |
|  | LysM-R | GCT AGA GCC TGT TTT GCA CGT TCA |
| *Arginase-1* | Arg1-F1 | GGAGAGCCTTCCTGCACTTT |
|  | Arg1-R1 | GTGCCTTGGTCTACATTGAACATAC |
| *Arginase-2* | Arg2-F | CCAGCTGCCATTCGAGAAG |
|  | Arg2-R | ATCATCTTGTGGGACATTAGTAAACTC |
| *Nos-2* | Nos2-F | GCCACCAACAATGGCAACA |
|  | Nos2-R | CGTACCGGATGAGCTGTGAATT |
| *Scl7a1* | Cat1-F | CTGGTGGACCTCATGTCCATT |
|  | Cat1-R | GCTCATTCTGATCTACTCGATCTAGCT |
| *Slc7a2b* | Cat2b-F | GATCCATTTTCCCAATGCCTC |
|  | Cat2b-R | TGGAATTGATTTGAGCTAGACATTTG |
| *Slc7a7* | Lat1-F | GAAGGACCCCGACCGG |
|  | Lat1-R | AACAGCCACCAGGAAGATGG |
| *Il4* | IL4-F | TGGAATGTACCAGGAGCCATATCC |
|  | IL4-R | CTCTGTGGTGTTCTTCGTTGCTGT |
| *Il13* | IL13-F | CACACAAGACCAGACTCCCCTG |
|  | IL13-R | GGTTACAGAGGCCATGCAATATCC |
| *Il5* | IL5-F | ATCAAACTGTCCGTGGGGGTACT |
|  | IL5-R | TCTCTCCTCGCCACACTTCTCTTT |
| *Il10* | IL10-F | GGACAACATACTGCTAACCGACTCCT |
|  | IL10-R | CTGCTCCACTGCCTTGCTCTTATT |
| *Ccl2* | MCP1-F | GCTGGAGAGCTACAAGAGGAT |
|  | MCP1-R | ACAGACCTCTCTCTTGAGCTTGGT |
| *Ccl11* | Eotaxin1-F | CTGCTGCTCACGGTCACTTCCT |
|  | Eotaxin1-R | CAGGGTGCATCTGTTGTTGGTG |
| *Ifng* | IFNG-F | GGTTGCTCCTCTTACCGTCTTT |
|  | INFG-R | CGTGGCACTTTTTACCACAGA |
| *Tnfa* | TNFA-F | TCAATCGGCCCGACTATCTC |
|  | TNFA-R | CAGGGCAATTGATCCCAAAGT |
| *Muc5ac* | MUC5AC-F | GATGACTTCCAGACTATCAGTG |
|  | MUC5AC-R | TGGCGTTAGTCAGCAGA |
| *Clca3* | CLCA3-F | CCGGCTGCCGCTAAAGAG |
|  | CLCA3-R | CAGAAGCATCAACAAGACCATTG |
| *18S* | 18S-F | AGTTAGCATGCCAGAGTCTCG |
|  | 18S-R | TGCATGGCCGTTCTTAGTTG |

**Cloots et al. Supplemental Table 2:** Amino-acid concentrations in venous plasma (µM ± SEM) of Arg1-Con and Arg1-KO^Tie2Cre^ mice *; P< 0.05 OVA/OVA vs. PBS/OVA Arg1-Con; #: P<0.05 OVA/OVA vs. PBS/OVA Arg1-KO^Tie2^.

|  | Arg1-Con | | Arg1-KO^Tie2^ | |
| --- | --- | --- | --- | --- |
| Amino acid | PBS/OVA | OVA/OVA | PBS/OVA | OVA/OVA |
| Glu | 23 ± 7 | 32 ± 4 | 23 ± 7 | 32 ± 3 |
| Asn | 48 ± 7 | 38 ± 3 | 53 ± 8 | 35 ± 3 |
| Ser | 112 ± 14 | 121 ± 17 | 133 ± 14 | 119 ± 13 |
| Gln | 903 ± 27 | 941 ± 46 | 1004 ± 51 | 899 ± 45 |
| His | 110 ± 6 | 108 ± 4 | 110 ± 9 | 106 ± 6 |
| Gly | 276 ± 40 | 243 ± 17 | 294 ± 20 | 210 ± 11 **#** |
| Thr | 156 ± 4 | 158 ± 8 | 151 ± 6 | 164 ± 10 |
| Cit | 89 ± 9 | 79 ± 4 | 78 ± 4 | 80 ± 3 |
| Arg | 134 ± 9 | 87 ± 9 ***** | 119 ± 10 | 105 ± 12 |
| Ala | 848 ± 70 | 839 ± 45 | 828 ± 69 | 799 ± 58 |
| Tyr | 160 ± 13 | 131 ± 9 | 168 ± 23 | 144 ± 22 |
| Tau | 240 ± 24 | 199 ± 6 | 239 ± 18 | 208 ± 6 |
| Val | 299 ± 12 | 277 ± 14 | 302 ± 18 | 280 ± 12 |
| Met | 54 ± 1 | 49 ± 1 | 57 ± 4 | 52 ± 3 |
| Ile | 179 ± 10 | 156 ± 9 | 175 ± 12 | 162 ± 5 |
| Phe | 52 ± 4 | 44 ± 3 | 49 ± 5 | 45 ± 3 |
| Orn | 42 ± 4 | 76 ± 8 ***** | 35 ± 5 | 70 ± 5 **#** |
| Leu | 225 ± 12 | 202 ± 12 | 221 ± 18 | 213 ± 7 |
| Trp | 321 ± 9 | 297 ± 16 | 287 ± 15 | 273 ± 17 |
| Lys | 532 ± 13 | 489 ± 17 | 490 ± 24 | 456 ± 24 |
| total AAs | 4628 ±167 | 4565 ± 170 | 4672 ± 294 | 4441 ± 175 |
| Arg/Orn | 3.2 ± 0.3 | 1.1 ± 1.2* | 3.4 ± 0.7 | 1.1 ± 0.1# |
| Arg/(Orn+Lys) | 0.22 ± 0.01 | 0.15 ± 0.01 | 0.21 ± 0.01 | 0.20 ± 0.02 |

**Cloots et al. Supplemental Table 3:** Differences between male and female Arg1-Con mice after treatment with the OVA/OVA protocol.

| **Parameter** | **Value** | **Arg1-Con female** | **Arg1-Con male** | **P value** |
| --- | --- | --- | --- | --- |
| H 0.0 mg/ml metacholine | cmH_2_O.s/ml | 27 ± 1 | 22 ± 1 | 0.0001 |
| H 3.12 mg/ml metacholine | cmH_2_O.s/ml | 35 ± 4 | 26 ± 2 | 0.0001 |
| H 12.5 mg/ml metacholine | cmH_2_O.s/ml | 43 ± 6 | 32 ± 2 | 0.002 |
| *Arg1* | AU | 416 ± 53 | 138 ± 36 | 0.007 |
| *Il4* | AU | 4 ± 1 | 1 ± 0 | 0.003 |
| *Ccl11* | AU | 77 ± 10 | 44 ± 5 | 0.001 |
| *Ifng* | AU | 81 ± 12 | 48 ± 3 | 0.005 |
| *Muc5ac* | AU | 28 ± 3 | 12 ± 2 | 0.009 |
| *Slc7a7* | AU | 19 ± 2 | 13 ± 1 | 0.002 |
| *Clca3* | AU | 597 ± 81 | 324 ± 52 | 0.031 |
| IFN-γ | pg/mg tissue | 16 ± 2 | 11 ± 2 | 0.063 |
| IL-10 | pg/mg tissue | 4 ± 0 | 6 ± 0 | 0.003 |
| IL-13 | pg/mg tissue | 59 ± 9 | 74 ± 4 | 0.034 |
| IgE | ng/ml plasma | 18 ± 3 | 6 ± 2 | 0.014 |

**Supplemental Figure 1. Correlation of lung-function, abundance of pulmonary mRNAs and proteins, pulmonary histopathology, and concentrations of plasma amino acids in wild-type female mice.** The lower-left triangle refers to data obtained female and the upper-right triangle to data from male Arg1-Con mice. The numbers show the correlation coefficients between parameters indicated above and to the left of the columns and rows, respectively, as measured in all 15 or 16 male and female Arg1-Con mice studied, that is, both the PBS/OVA- and the OVA/OVA-treated groups. The significance of the correlations is color-coded according to the P-value of the correlation coefficient: yellow: 0.05>P>0.01, orange: 0.01>P>0.001, and red: P<0.001. Note the difference in the degree of cross-correlations of parameters between the categories named in the title in males and females.
