# Supplementary material for: Arginase 1 deletion in myeloid cells affects the inflammatory response in allergic asthma, but not lung mechanics, in female mice
Source: BMC Pulm Med. 2017 Nov 28;17:158. doi: 10.1186/s12890-017-0490-7 (PMC5706166; doi:10.1186/s12890-017-0490-7)
Supplement: Supplementary file 2 — Correlation of lung-function, abundance of pulmonary mRNAs and proteins, pulmonary histopathology, and concentrations of plasma amino acids in wild-type male and female mice. The lower-left triangle refers to data obtained female and the upper-right triangle to data from male Arg1-Con mice. The numbers show the correlation coefficients between parameters indicated above and to the left of the columns and rows, respectively, as measured in all 15 or 16 male and female Arg1-Con mice studied, that is, both the PBS/OVA- and the OVA/OVA-treated groups. The significance of the correlations is color-coded according to the P-value of the correlation coefficient: yellow: 0.05 > P > 0.01, orange: 0.01 > P > 0.001, and red: P < 0.001. Note the difference in the degree of cross-correlations of parameters between the categories named in the title in males and females. (PDF 219 kb) [file 12890_2017_490_MOESM2_ESM.pdf]

Arg1-Con

Correlations female Arg1-Con and male Arg1-Con mice		Lung function												mRNA expression																Protein Expression									Histology		Plasma [AA]			
		Rn max sal	Rn max 3.1	Rn max 12.5	Rn max 50	H max sal	H max 3.1	H max 12.5	H max 50	G max sal	G max 3.1	G max 12.5	G max 50	*Slc7a7*	*Slc7a1*	*Slc7a2b*	*Arg1*	*Arg2*	*Nos2*	*Il4*	*Il13*	*Il5*	*Ccl11*	*Il10*	*Ccl2*	*Muc5ac*	*Clc3a*	*Tnfa*	*Ifng*	ARG1	IL4	IL13	IL5	CCL11	TNFα	IL10	IFNγ	Ige OVA	MBP	MPO	[Arg]	[Cit]	[Orn]	Σ[AA]
Lung function	Rn max sal		0.771	0.61	0.284	0.676	0.535	0.658	0.395	0.675	0.706	0.68	0.606	-0.03	0.38	0.123	0.246	0.118	-0.04	0.48	0.446	0.214	0.337	0.272	0.293	0.432	0.506	0.476	0.048	0.24	0.119	-0.1	0.171	0.169	0.074	-0.31	0.125	-0.01	0.063	0.29	-0.38	-0.22	0.383	-0.18
	Rn max 3.1	.453		0.832	0.338	0.473	0.374	0.407	0.3	0.547	0.65	0.548	0.682	0.247	0.603	0.145	0.386	0.204	0.107	0.355	0.372	0.269	0.369	0.449	0.346	0.512	0.384	0.57	0.146	0.036	0.049	-0.15	0.077	0.051	-0.07	-0.24	-0.02	0.147	-0.03	0.569	-0.21	-0.13	0.077	-0.33
	Rn max 12.5	.481	.730		0.792	0.532	0.393	0.466	0.672	0.546	0.656	0.743	0.921	0.307	0.491	0.256	0.535	0.36	0.222	0.531	0.349	0.359	0.428	0.539	0.453	0.615	0.429	0.642	0.048	0.62	0.251	-0.05	0.236	0.252	0.185	-0.31	0.186	0.249	-0.03	0.481	-0.26	-0.06	0.107	-0.39
	Rn max 50	.726	.625	.780		0.836	0.672	0.79	0.826	0.624	0.817	0.832	0.895	0.263	0.235	0.358	0.605	0.493	0.509	0.76	0.31	0.412	0.364	0.45	0.441	0.526	0.397	0.57	-0.29	0.915	0.46	0.084	0.323	0.461	0.824	-0.33	0.379	-0.04	0.111	0.423	-0.26	0.014	0.119	0.153
	H max sal	.487	.434	.461	.453		0.806	0.941	0.877	0.905	0.873	0.892	0.898	0.051	0.459	0.229	0.466	0.277	0.266	0.915	0.567	0.343	0.375	0.452	0.469	0.588	0.606	0.664	-0.27	0.63	0.484	0.078	0.31	0.521	0.589	-0.48	0.393	0.421	0.125	0.517	-0.28	-0.05	0.467	0.179
	H max 3.1	.383	.359	.521	.374	.914		0.814	0.749	0.894	0.882	0.853	0.926	-0.04	0.329	0.416	0.48	0.313	0.287	0.74	0.777	0.568	0.303	0.457	0.401	0.434	0.425	0.344	-0.37	0.781	0.316	0.4	0.418	0.61	0.491	0.099	0.109	-0.53	0.369	0.266	-0.11	-0.13	0.302	0.262
	H max 12.5	.390	.387	.673	.456	.765	.932		0.972	0.882	0.842	0.862	0.844	-0.21	0.178	0.025	0.237	0.012	0.066	0.846	0.373	0.161	0.11	0.226	0.213	0.357	0.391	0.603	-0.48	0.606	0.544	0.321	0.493	0.63	0.507	-0.23	0.494	0.364	0.111	0.258	-0.17	-0.03	0.494	0.206
	H max 50	.419	.307	.700	.677	.635	.656	.735		0.77	0.711	0.865	0.832	0.185	0.183	0.382	0.569	0.456	0.354	0.869	0.414	0.473	0.44	0.529	0.527	0.573	0.483	0.614	-0.26	0.887	0.531	0.395	0.608	0.737	0.523	-0.18	0.467	0.324	0.221	0.331	-0.42	-0.09	0.449	-0.21
	G max sal	.160	.282	.186	.013	.707	.584	.390	.302		0.844	0.822	0.81	-0.07	0.395	0.115	0.36	0.18	0.036	0.765	0.545	0.334	0.249	0.422	0.374	0.463	0.402	0.513	-0.38	0.726	0.432	0.233	0.394	0.537	0.264	-0.36	0.388	0.331	-0.16	0.444	-0.14	0.028	0.327	0.203
	G max 3.1	.043	.550	.415	.098	.732	.768	.654	.338	.759		0.912	0.92	0.004	0.531	0.106	0.374	0.185	0.124	0.642	0.384	0.289	0.192	0.369	0.318	0.478	0.393	0.648	-0.32	0.531	0.151	-0.08	0.074	0.186	0.433	-0.36	0.135	0.6	-0.02	0.452	-0.03	0.105	0.08	0.111
	G max 12.5	.369	.564	.793	.476	.631	.738	.849	.727	.449	.660		0.951	0.048	0.378	0.233	0.469	0.309	0.17	0.718	0.415	0.391	0.312	0.437	0.429	0.57	0.507	0.636	-0.27	0.818	0.35	-0.01	0.227	0.351	0.518	-0.44	0.361	0.674	0.029	0.367	-0.2	0.02	0.234	0.002
	G max 50	.216	.312	.686	.455	.332	.497	.699	.742	.075	.304	.843		0.46	0.561	0.508	0.739	0.623	0.408	0.751	0.536	0.627	0.538	0.706	0.642	0.744	0.566	0.759	0.052	0.981	0.353	-0.07	0.173	0.334	0.481	-0.52	0.314	0.517	0.042	0.533	-0.27	0.054	0.119	-0.32
mRNA expression	*Slc7a7*	.216	.205	.012	.103	.378	.103	-.067	.064	.552	.193	.047	-.358		0.89	0.581	0.718	0.804	0.713	0.685	0.564	0.547	0.708	0.734	0.73	0.68	0.56	0.795	0.728	-0.21	-0.09	-0.42	-0.3	-0.12	0.146	-0.48	-0.36	0.26	0.049	0.486	-0.22	-0.05	-0.08	-0.27
	Slc7a1	-.031	-.033	-.163	-.146	.295	.005	-.247	-.006	.654	.272	-.104	-.454	.843		0.523	0.734	0.756	0.62	0.687	0.595	0.559	0.733	0.805	0.777	0.794	0.665	0.878	0.717	-0.2	-0.24	-0.38	-0.29	-0.14	0.009	-0.38	-0.41	0.241	0.098	0.653	-0.09	0.117	-0.04	-0.17
	Slc7a2b	.633	.394	.167	.293	.704	.550	.442	.352	.449	.357	.269	.063	.394	.251		0.879	0.895	0.717	0.766	0.955	0.963	0.792	0.833	0.809	0.653	0.637	0.405	0.391	0.555	0.193	0.055	0.033	0.37	0.448	-0.16	0.024	-0.59	0.633	0.398	-0.39	-0.17	0.191	-0.17
	Arg1	.745	.538	.295	.472	.453	.297	.282	.273	.139	.058	.343	.127	.434	.175	.759		0.913	0.842	0.933	0.888	0.905	0.818	0.951	0.896	0.787	0.688	0.714	0.389	0.668	0.308	-0.03	0.014	0.336	0.519	-0.32	0.078	-0.19	0.397	0.634	-0.31	-0.02	0.182	-0.11
	Arg2	.737	.501	.225	.445	.619	.409	.286	.309	.377	.163	.147	-.066	.546	.336	.898	.749		0.727	0.935	0.9	0.878	0.887	0.9	0.931	0.777	0.7	0.598	0.563	0.297	0.089	-0.13	-0.04	0.224	0.399	-0.36	-0.11	-0.29	0.245	0.517	-0.34	-0.06	0.021	-0.2
	Nos2	.547	.362	.196	.336	.635	.495	.455	.336	.248	.249	.363	.064	.520	.226	.779	.857	.690		0.773	0.642	0.696	0.583	0.715	0.651	0.543	0.498	0.713	0.38	0.565	0.286	-0.13	-0.11	0.192	0.62	-0.29	0.029	-0.02	0.432	0.462	-0.21	0.042	0.139	0.125
	Il4	.573	.383	.273	.255	.785	.723	.719	.499	.515	.538	.628	.573	.159	.087	.975	.651	.850	.682		0.797	0.788	0.839	0.857	0.904	0.793	0.765	0.709	0.345	0.921	0.486	0.075	0.466	0.671	0.605	-0.41	0.291	0.029	0.155	0.651	-0.47	-0.07	0.421	0.027
	Il13	.397	.243	.222	.155	.725	.662	.674	.520	.542	.551	.632	.617	.226	.182	.922	.534	.746	.621	.960		0.982	0.865	0.894	0.876	0.726	0.72	0.428	0.381	0.707	0.204	0.143	0.16	0.439	0.322	-0.13	0.009	-0.49	0.553	0.496	-0.43	-0.22	0.304	-0.12
	Il5	.463	.388	.100	.034	.682	.675	.680	.274	.484	.604	.596	.303	.299	.112	.883	.664	.702	.749	.862	.825		0.8	0.879	0.838	0.674	0.623	0.428	0.312	0.636	0.231	0.188	0.155	0.449	0.411	-0.05	0.017	-0.6	0.577	0.466	-0.34	-0.17	0.193	-0.13
	Ccl11	.453	.269	.134	.182	.735	.580	.484	.411	.522	.467	.412	.127	.521	.372	.885	.759	.715	.906	.872	.873	.843		0.855	0.949	0.887	0.886	0.62	0.588	0.596	0.129	-0.11	0.154	0.352	0.27	-0.47	0.029	-0.11	0.399	0.685	-0.61	-0.27	0.316	-0.36
	Il10	.506	.367	.456	.462	.609	.507	.622	.607	.303	.223	.622	.721	.470	.240	.816	.689	.711	.812	.754	.753	.624	.857		0.946	0.852	0.723	0.689	0.448	0.686	0.163	0.038	0.04	0.336	0.261	-0.25	-0.05	0.032	0.327	0.666	-0.31	-0.03	0.173	-0.22
	Ccl2	.441	.309	.313	.324	.516	.302	.236	.560	.495	.273	.392	.276	.476	.501	.709	.622	.626	.566	.709	.778	.463	.772	.725		0.893	0.838	0.662	0.531	0.639	0.14	-0.03	0.064	0.332	0.294	-0.37	-0.02	-0.02	0.315	0.681	-0.44	-0.11	0.223	-0.25
	Muc5ac	.341	.254	.193	.299	.547	.404	.385	.573	.387	.316	.437	.251	.522	.398	.632	.708	.483	.828	.591	.585	.603	.845	.802	.681		0.928	0.744	0.396	0.696	0.044	-0.18	0.134	0.327	0.234	-0.55	-0.03	0.406	0.35	0.765	-0.54	-0.17	0.321	-0.34
	Clc3a	.462	.326	.198	.294	.606	.457	.417	.532	.424	.353	.453	.240	.516	.375	.768	.818	.615	.882	.735	.709	.747	.918	.835	.741	.972		0.665	0.416	0.668	0.075	-0.22	0.134	0.33	0.288	-0.62	0.063	0.265	0.501	0.655	-0.69	-0.27	0.521	-0.3
	Tnfa	.026	.160	.241	.166	.245	.174	.241	.443	.316	.226	.421	.389	.586	.556	.189	.177	.262	.276	.085	.293	.315	.288	.347	.298	.482	.423		0.617	0.103	0.093	-0.33	-0.01	0.191	0.277	-0.46	-0.02	0.718	0.206	0.571	-0.31	0.127	0.355	-0.19
	Ifng	-.169	-.251	-.385	-.242	-.237	-.448	-.642	-.462	.201	-.240	-.517	-.676	.607	.666	-.249	-.146	.039	-.214	-.630	-.545	-.472	-.243	-.413	-.109	-.186	-.218	.209		-0.72	-0.39	-0.49	-0.49	-0.42	-0.26	-0.27	-0.51	0.137	0.043	0.239	-0.2	-0.04	-0.1	-0.38
Protein Expression	ARG1	.384	.132	.050	.138	.651	.753	.952	.441	-.259	.226	.634	.501	.016	-.631	.660	.775	.430	.977	.595	.656	.764	.934	.721	.437	.837	.919	.335	-.869		0.547	0.724	0.744	0.889	0.968	0.287	0.342	-0.91	0.866	0.653	-0.64	-0.53	0.59	0.259
	IL4	.285	.253	.317	.326	.450	.367	.467	.588	.376	.296	.495	.659	.243	.174	.678	.400	.472	.535	.681	.751	.506	.726	.822	.726	.729	.723	.245	-.567	.611		0.415	0.447	0.588	0.645	-0.15	0.817	-0.26	0.097	0.351	-0.38	-0.36	0.483	0.053
	IL13	.591	.594	.528	.505	.709	.656	.761	.749	.477	.486	.786	.742	.352	.171	.770	.561	.633	.690	.747	.762	.752	.778	.773	.637	.738	.776	.385	-.551	.855	.807		0.673	0.701	0.008	0.677	0.307	-0.16	0.263	-0.05	-0.07	-0.23	0.247	-0.1
	IL5	.226	.266	.273	.136	.794	.761	.716	.611	.738	.789	.680	.633	.261	.364	.724	.248	.538	.424	.821	.900	.647	.742	.568	.717	.472	.544	.207	-.485	.478	.681	.703		0.897	0.149	0.12	0.512	0.027	0.221	0.021	-0.54	-0.38	0.557	-0.22
	CCL11	.398	.385	.422	.370	.758	.723	.822	.724	.414	.518	.731	.790	.326	.159	.824	.493	.667	.738	.818	.881	.712	.870	.853	.694	.697	.738	.329	-.674	.863	.795	.844	.826		0.377	0.043	0.599	-0.02	0.486	0.207	-0.61	-0.32	0.669	-0.16
	TNFα	.632	.555	.423	.428	.627	.582	.678	.625	.380	.375	.609	.671	.259	.086	.902	.621	.810	.639	.867	.852	.824	.753	.791	.622	.634	.730	.373	-.559	.699	.757	.891	.673	.826		-0.37	0.581	-0.43	0.313	0.289	-0.29	-0.04	0.223	0.296
	IL10	.441	.372	.331	.479	.138	.147	.284	.299	-.037	-.029	.211	.315	-.188	-.415	.275	.167	.192	.194	.342	.289	.246	.168	.209	.078	.181	.198	-.133	-.464	.696	.508	.572	.152	.287	.467		-0.28	-0.35	0.142	-0.48	0.475	0.003	-0.35	0.117
	IFNγ	-.122	-.157	-.012	.206	-.274	-.256	-.079	.268	-.356	-.377	-.136	.232	-.136	-.260	-.072	-.064	-.125	.057	-.067	.003	-.153	-.005	.198	-.005	.330	.192	.196	-.299	.440	.473	.217	-.126	.182	.199	.458		-0.11	0.182	0.207	-0.45	-0.11	0.592	-0.09
	Ige OVA	.121	.680	.705	-.010	-.077	.111	.301	.121	.202	.335	.693	.292	.822	.620	.286	.739	.241	.429	.266	.200	.336	.845	.196	.646	.649	.851	.308	.856	.277	.049	.315	-.051	-.569	.155	-.398	-.305		-0.32	0.221	-0.21	0.377	0.331	-0.37
Histology	MBP	.818	.735	.547	.647	.914	.816	.686	.754	.191	.411	.622	.464	.205	.043	.709	.812	.621	.781	.741	.643	.668	.753	.876	.701	.756	.781	.121	-.462	.933	.708	.857	.799	.917	.758	.259	-.010	-.573		0.222	-0.53	-0.43	0.556	-0.26
	MPO	.594	.556	.417	.437	.621	.543	.506	.639	.124	.250	.435	.185	.322	.156	.492	.504	.441	.613	.368	.390	.538	.553	.613	.455	.605	.585	.416	-.229	.997	.565	.882	.578	.835	.696	.435	.317	-.030	.760		-0.34	-0.21	0.133	-0.22
Plasma [AA]	[Arg]	-.181	-.187	-.152	-.062	-.519	-.455	-.391	-.274	-.462	-.447	-.442	-.082	-.431	-.253	-.271	-.324	-.201	-.554	-.211	-.263	-.306	-.565	-.286	-.388	-.496	-.477	-.023	.034	-.536	-.281	-.430	-.403	-.422	-.110	-.110	.118	-.158	-.439	-.405		0.618	-0.72	0.488
	[Cit]	.147	-.004	-.242	-.178	-.140	-.288	-.366	-.229	.077	-.180	-.101	-.238	.435	.441	.067	.446	.142	.196	.020	-.035	.203	.128	.047	.170	.382	.395	.411	.387	-.283	-.097	.058	-.203	-.203	.104	-.213	-.032	.061	.047	.050	.153		-0.32	0.341
	[Orn]	-.384	-.066	-.172	-.289	-.054	-.062	-.100	-.162	.043	.193	-.131	-.208	.148	.230	.053	-.024	-.061	.082	-.056	-.065	.113	.133	.024	.028	.229	.171	-.050	-.088	.223	.336	.028	.164	.268	.075	-.181	.352	.183	.297	.173	-.013	-.015		-0.18
	Σ[AA]	-.276	.032	-.141	-.263	-.414	-.311	-.267	-.300	-.261	-.064	-.199	-.127	-.321	-.180	-.308	-.200	-.395	-.333	-.275	-.394	-.189	-.362	-.374	-.352	-.194	-.223	-.290	-.193	-.296	-.188	-.267	-.407	-.440	-.221	-.004	.188	.461	-.141	-.182	.335	.187	.661	

Arg1-Con
